# Ensemble Deep Learning for Biomedical Time Series Classification

**DOI:** 10.1155/2016/6212684

**Published:** 2016-09-20

**Authors:** Lin-peng Jin, Jun Dong

**Affiliations:** ^1^Suzhou Institute of Nanotech and Nanobionics, Chinese Academy of Sciences, Suzhou 215123, China; ^2^University of Chinese Academy of Sciences, Beijing 100049, China

## Abstract

Ensemble learning has been proved to improve the generalization ability effectively in both theory and practice. In this paper, we briefly outline the current status of research on it first. Then, a new deep neural network-based ensemble method that integrates filtering views, local views, distorted views, explicit training, implicit training, subview prediction, and Simple Average is proposed for biomedical time series classification. Finally, we validate its effectiveness on the Chinese Cardiovascular Disease Database containing a large number of electrocardiogram recordings. The experimental results show that the proposed method has certain advantages compared to some well-known ensemble methods, such as* Bagging* and* AdaBoost*.

## 1. Introduction

In the field of pattern recognition, the target of classification is to construct a decision-making function in nature. We can obtain the best ones via simple calculation for linear classification problems, while it is not easy to determine the best ones for nonlinear classification problems, such as image recognition and biomedical time series classification. The processing flow of traditional methods is such that feature vectors are extracted from raw data first (feature selection is conducted when necessary), and then a suitable model based on them is employed for classification. If we let *f*(*x*) and *g*(*x*) be the corresponding function of these two parts, the constructed decision-making function can be written as *g*(*f*(*x*)). However, with many of the existing feature extraction technologies and classification algorithms, we cannot have highly complicated nonlinear functions. For example, principal component analysis and independent component analysis are both linear dimensionality reduction algorithms, the wavelet transformation is a simple integral transformation, and the Gaussian mixture model is made of a finite number of Gaussian functions. Therefore, many traditional methods do not perform very well in hard artificial intelligence task.

**Figure 1:**
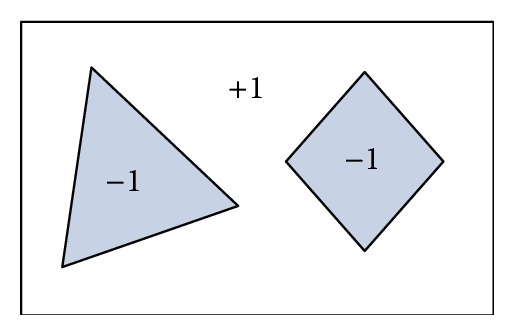
Ensemble learning.

Achieving great success in complicated fields of pattern recognition in recent years, deep learning [1, 2] is a deep neural network (DNN) with more than 3 layers, which inherently fuses “feature extraction” and “classification” into a signal learning body and directly constructs a decision-making function. Obviously, its ability to construct nonlinear functions becomes strong with the increasing number of layers and neurons, but the number of network weights that need to be adjusted is significantly increased. On the other hand, with ensemble learning that combines multiple classifiers [3], we can also have complicated decision-making functions. As shown in Figure 1, we can obtain a nonlinear classification model via seven linear classifiers (the filled and unfilled regions denote two classes, resp.). In fact, the constructive mechanism of ensemble learning is the same as that of support vector machine, which constructs nonlinear functions by combining multiple kernel functions.

The pioneering work of ensemble learning was done in 1990 [4, 5], which proved that multiple weak learning algorithms could be converted into a strong learning algorithm in theory, and since then many scholars have carried out widespread and thorough research. In general, ensemble-learning algorithms consist of two parts: how to generate differentiated individual classifiers and how to fuse them, namely, generation strategies and fusion strategies. Next, we will provide a brief review of both of them.

There are two kinds of generation strategies, namely, the heterogeneous type and the homogeneous type. The former is such that individual classifiers are generated using different learning algorithms. We will not elaborate on this type since it is relatively simple. The latter uses the same learning algorithm, so different settings (such as learning parameters and training samples) are necessary. Many methods have been developed for this subject and can be divided into four categories.

The first way is to manipulate training samples. For instance,* Bagging* [7] creates multiple data sets by sampling with replacement from the original training samples, each of which is used to train an individual classifier.* Boosting* [8–10] is another example, in which the learning algorithm uses a different weighting or distribution over the training samples at each iteration, according to the errors of the individual classifiers. There are also other approaches, such as* Cross-Validated Committees* [11],* Wagging* [12], and* Arcing* [13]. The second way is to manipulate input features.* Random Subspace *[14] generates different randomly selected subsets of input features and trains an individual classifier on each of them, while* Input Decimation* [15] trains the individual classifier only on the most correlated subset of input features for each class. There are also other methods, such as* Rotation Forest* [16] and* Similarity-Based Feature Space* [17]. The third way is to manipulate class labels. For instance,* Output Coding* [18–20] decomposes a multiclassification task into a series of binary-classification subtasks and trains individual classifiers for them.* Class-Switching *[21, 22] is another example, which generates an individual classifier based on randomly changing the class label of a fraction of the training samples. The last way is to inject randomness into the learning algorithm. For example, using the backpropagation (BP) algorithm, the resulting classifiers can be quite different if neural networks with different initial weights are applied to the same training samples [23]. There are also other approaches such as* Randomized First-Order Inductive Learner* [24] and* Random Forest* [25].

In a word, the core of generation strategies is to make individual classifiers different (independent errors and diversity), and only when this condition is satisfied can the classification performance be improved; that is, a good decision-making function can be constructed. As for fusion strategies,* Major Voting *[26] is one of the most popular methods, in which each individual classifier votes for a specific class, and the predicted class is the one that collects the largest number of votes.* Simple Average *and* Weighted Average* [27] are also commonly used. Besides them, one can also employ other methods to combining individual classifiers, such as* Dempster-Shafer Combination Rules* [28],* Stacking Method* [29], and* Second-Level Trainable Combiners* [30].

Since both deep learning and ensemble learning have advantages in constructing complicated nonlinear functions, the combination of the two can better handle hard artificial intelligence tasks. Deng and Platt [31] adopted linear and log-linear stacking methods to fuse convolutional, recurrent, and fully connected DNNs. Xie et al. [32] proposed three DNN-based ensemble methods, that is, “fusing a series of classifiers whose inputs are the representation of intermediate layers” and “using* Major Voting* and* Stacking Method* to fuse a series of classifiers obtained within a relatively stable range of epoch.” Zhang et al. [33] presented several methods for integrating* Restricted Boltzmann Machines* with* Bagging* to construct multiple individual classifiers. Qiu et al. [34] employed a model of* Support Vector Regression* (*Stacking Method*) to aggregate the outputs from various deep belief networks. Zhang et al. [35] trained an ensemble of DNNs whose initial weights were initialized differently and penalizes the differences between the output of each DNN and their average output. Huang et al. [36] presented an ensemble criterion of DNNs based on the reconstruction error. In conclusion, we can use many existing strategies to construct a good ensemble of DNNs, such as setting different architectures, injecting noise, and employing the framework of* AdaBoost* [37–41].

In this paper, we propose a novel DNN-based ensemble method for biomedical time series classification. Based on the local and distorted view transformations, different types of digit filters and different validation mechanisms are used to generate individual classifiers, and “subview prediction” and “Simple Average” are utilized to fuse them. In what follows, Section 2 presents our proposed method in detail. In Sections 3 and 4, the experimental step is described and the experimental results are reported.  Section 5 concludes the paper.

## 2. Methodologies

**Figure 2:**
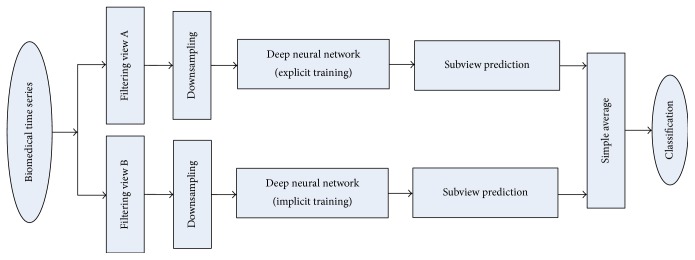
Overview of the proposed method.

Figure 2 depicts the full process of the proposed method. First, we utilize different filtering methods to preprocess the biomedical time series and selectively conduct downsampling operation and then respectively employ the explicit method and the implicit method to train two DNNs. In the testing phase, we independently apply “subview prediction” to two DNNs first, and then use “*Simple Average*” to incorporate the outputs of them.

### 2.1. Filtering View

In practical application, collected biomedical time series are often contaminated by interfering noise. Although we can perform denoising, some useful information may be lost after doing that. DNNs have the ability to capture useful information but ignore interfering noise after learning from a certain number of training samples, and an effective strategy for homogeneous ensemble learning is to make input data different. Therefore, we just extract different view data from raw biomedical time series.

### 2.2. Deep Neural Network

At present, DNNs mainly include convolutional neural networks (CNNs) [42], deep belief networks [43], and stacked denosing autoencoders [44], among which CNNs utilize “weight sharing” and “pooling” to make the number of weights not increase dramatically when the numbers of input neurons and layers are very large, so that they can be widely used in various fields of pattern recognition. However, as a model developed for images, CNNs perform convolution operations in both horizontal and vertical directions. It is a reasonable thing to do since image data are relevant in both directions. However, for biomedical time series with multiple channels (also organized as a matrix), directly employing CNNs for classification is not very appropriate since the data in the horizontal direction (intrachannel) are relevant while the data in the vertical direction (interchannel) are independent. For this, the previous works [6, 45, 46] developed multichannel convolutional neural networks (MCNNs) which possess better classification performance.

**Figure 3:**
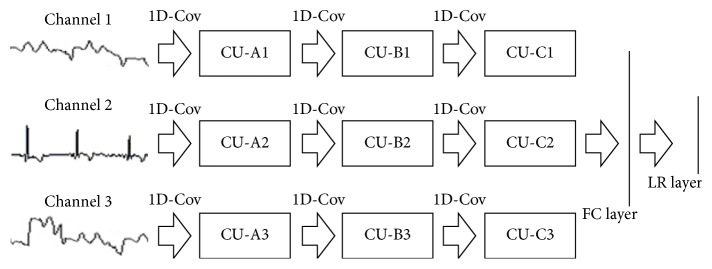
Architecture of MCNN.

An example of 3-stage MCNN is shown in Figure 3: data of each channel go through three different convolution units (CU) first, and then information from all the channels is inputted into a fully connected (FC) layer; finally, the predictive value is outputted by a logistic regression (LR) layer. Note that a CU consists of a convolutional layer and a subsampling layer (max-pooling layer) and “1D-Cov” denotes a one-dimensional convolution operation.

### 2.3. Explicit Training

The DNN can construct a nonlinear function when each network weigh is assigned a value. Obviously, the number of constructed nonlinear functions becomes large with the increasing number of weights. The nature of network training is to determine which function is the best for a given problem. As a most common used method, the BP algorithm cannot find out a good nonlinear function unless there are enough training samples. It does not mean to increase the size of training set really but to increase the number of training samples presented to the network. Fortunately, the virtual sample technology is up to this task, whose core is to perform a transformation on biomedical time series under the premise of preserving class labels. There are mainly the local view transformation and the distorted view transformation. The former is to extract subseries and the latter is to add distortion (e.g., adding random data with low amplitude or burst one).

Generally speaking, “explicit training” is adopted to train a DNN in supervised learning; that is, besides training samples, there are a small number of independent validation samples used to evaluate the obtained model during the training phase. Suppose the size of the biomedical time series is *C* × *F*1 (*C* is the number of channels;* F*1 is the number of data points in each channel) and the number of input neurons is *C* × *F*2 (*F*2 <* F*1); the training process can be described as follows: a random *C* × *F*2 subseries is extracted from the *C* × *F*1 training sample first (the local view transformation), and then it is added with some type of distortion with high probability (the distorted view transformation); finally, backpropagation is invoked. When* PMax* training samples have been presented to the network, the weights will be adjusted and the current DNN model will be tested by particular *C* × *F*2 subseries extracted from validation samples. If the accuracy (or other metrics) is the highest up to the present, the current DNN model will be saved. Afterwards, the next training sample is chosen to repeat the process.  Algorithm 1 gives the pseudocode of this training method.

### 2.4. Implicit Training

In “explicit training,” the DNN model is tested by validation samples at regular intervals in order to avoid overfitting, which also means that the selection of validation samples will significantly influence the classification performance. However, handpicking representative samples is not easy and limited by practical conditions in some case. For this, we develop “implicit training” in this study. As shown in Algorithm 2, most of the steps are the same as those shown in Algorithm 1; the main difference lies in the validation mechanism. When* PMax* training samples have been presented to the network, the weights will be adjusted and the current DNN model will be tested by particular *C* × *F*2 subseries extracted from the training samples, which is used between two adjacent weight-updating processes. At the end of every training epoch, the DNN model will be saved if the total accuracy (or other metrics) is the highest up to the present.

Someone may think that this method will result in overfitting, but this is a false judgment. For training, we extract random subseries and add distortion to them with high probability; and for validation, we extract particular subseries and do not add distortion to them. Hence, the probability of overlap is very small. Of course, we can use such validation mode only in the situation where the virtual sample technology is used.

### 2.5. Subview Prediction

The output of the DNN is a probability value that ranges from 0 to 1. If the value is about 0.5, the classification confidence is low. On the other hand, in the training phase, we utilize the virtual sample technology including the local view transformation and the distorted view transformation (collectively called the view transformation) to increase the number of training samples presented to the network. With these considerations in mind, we develop a new testing method called “subview prediction.”

**Figure 4:**
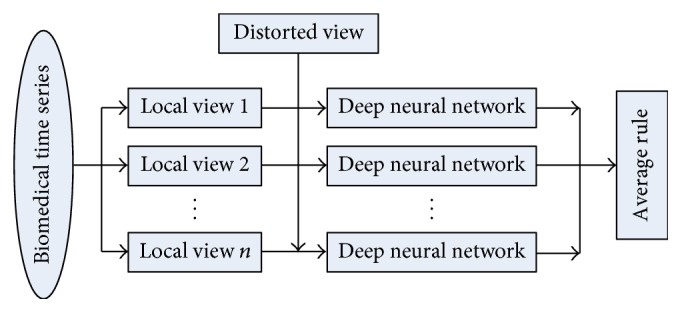
Diagram of subview prediction.

As shown in Figure 4, the testing process can be described as follows: *nC* × *F*2 subseries which start from *n* different positions are, respectively, extracted from the *C* × *F*1 testing sample first, and then they are selectively added with a type of distortion used in the training phase; finally, their predictive values outputted by the DNN are aggregated by the average rule (for the sake of simplicity). Note that only one *C* × *F*2 subseries is extracted and tested by the DNN in “simple prediction.”

### 2.6. Simple Average

*Simple Average* [27] is employed to fuse two DNNs in this paper: given* M*-class data, the predicted class *m* is determined by

(1) $$\begin{array}{c} m=arg\;\underset{1\leq i\leq 2}{max}\left\{\;\frac{1}{2}\overset{2}{\underset{j=1}{\sum }}P\left(\;y=i\;|\;c_{j}\;\right)\;\right\}, \end{array}$$

where *P*(*y* = *i*∣*c*_*j*_), *j* = 1,2, is the probability value predicted on the *i*th class by the DNN *c*_*j*_. Of course, we can use other fusion methods, such as* Weighted Average* [27],* Dempster-Shafer Combination Rules* [28], and* Second-Level Trainable Combiners* [30].

## 3. Experimental Setup

In this study, we apply the proposed ensemble method to one biomedical application, that is, classification of normal and abnormal electrocardiogram (ECG) recordings with short duration. It is useful for telemedicine centers where abnormal recordings are delivered to physicians for further interpretation after computer-assisted ECG analysis algorithms filter out normal ones, so that the diagnostic efficiency will be greatly increased [47]. However, this classification task is rather hard due to wild variations in ECG characteristics among different individuals. Many traditional methods do not perform well for this subject [48, 49]. In the research work [6], “low-pass filtering” and “downsampling (from original 500 Hz to 200 Hz)” are successively applied to ECG recordings first, and then one MCNN model is obtained by “explicit training.” By testing 151,274 recordings from the Chinese Cardiovascular Disease Database (CCDD) [50], it achieved the best results up to now. To ensure a fair comparison, the same ECG dataset is used to evaluate the performance of the newly proposed method.

### 3.1. ECG Dataset

The CCDD database consists of 179,130 standard 12-lead ECG recordings with sampling frequency of 500 Hz. After throwing away exception data, we choose 175,943 recordings for the numerical experiments where the numbers of training samples, validation samples, and testing samples (nine groups obtained from different sources) are 12320, 560, and 163063, respectively. Note that training samples and validation samples will be combined together in “implicit training.” These recordings are first processed using a digit filter, and then a downsampling operation (from original 500 Hz to 200 Hz) is conducted. Finally, 8 × 1900 sampling points are available for each recording. The Appendix shows the details of the process [6].

### 3.2. Individual Classifier

To ensure a fair comparison, all ensemble methods in the numerical experiments employ MCNNs as individual classifiers. In this study, two different network architectures, namely, MCNN[A] and MCNN[B], are used for different purposes. The first one is a 3-stage MCNN whose parameters are set as follows: the number of the neurons in the input layer is 8 × 1700 (1 × 1700); the sizes of three convolution kernels are 1 × 21, 1 × 13, and 1 × 9 (1 denotes the size in the vertical direction; 21 denotes the size in the horizontal direction), respectively; the sizes of three subsampling steps are 1 × 7, 1 × 6, and 1 × 6, respectively; the numbers of three feature maps are 6, 7, and 5, respectively; the numbers of the neurons in the FC layer and the LR layer are 50 and 1, respectively. The second one is also a 3-stage MCNN, whose parameters are set as follows: the number of the neurons in the input layer is 1 × 1900; the sizes of three convolution kernels are 1 × 18, 1 × 12, and 1 × 8, respectively; the sizes of three subsampling steps are 1 × 7, 1 × 6, and 1 × 6, respectively; the numbers of three feature maps are 6, 7, and 5, respectively; the numbers of the neurons in the FC layer and the LR layer are 50 and 1, respectively.

The local view transformation can be used for MCNN[A], but it cannot be used for MCNN[B] since the number of sampling points of an ECG recording is 1 × 1900. As for the distorted view transformation, it can be used for both MCNN[A] and MCNN[B]. In this study, random data with low amplitude (the maximal amplitude is lower than 0.15) is added to ECG recordings during the training phase, and this operation (e.g., the distorted view transformation) is ignored in the testing phase for the sake of simplicity.

Using MCNN[A], 8 × 1700 local segments which start from the 1st sampling point are extracted (from validation samples in “explicit training” and from training samples in “implicit training”) to evaluate the obtained model during the training phase. And in the testing phase, nine 8 × 1700 local segments which start from the 1st, 26th, 51st, 76th, 101st, 126th, 151st, 176th, and 201st sampling points are extract from a given ECG recording if “subview prediction” is used; otherwise, only one 8 × 1700 local segment which starts from the 1st sampling point is extracted. Using MCNN[B], we can train and test neural networks in a traditional manner.

We only employ the BP algorithm of inertia moment and variable step [51] in supervised learning and its related parameters are set as follows: the initial step size is 0.02, the step decay is 0.01 except for the 2nd epoch and the 3rd epoch (set as 0.0505),* PMax* is 560, and the maximal number of training epochs is 500. The experimental platform is based on an Intel Core i7-3770 CPU @3.4 GHz, 8.0 G RAM, 64-bit Window 7 operating system, and the* theano-0.6rc* [52] implementation is used.

### 3.3. Ensemble Method

*Bagging* [7] and* AdaBoost* [8] are two of the most popular and effective ensemble-learning methods, and they work especially well for unstable learning algorithms (i.e., small changes in the training data lead to large changes in the individual classifiers), such as neural networks and decision tress [53]. In addition, Ye et al. [54] proposed an ensemble method for multilead ECG that has excellent classification performance. To demonstrate the effectiveness of our proposed method, we compare it with these three ensemble methods. Next, we will provide their configuration parameters in detail:

1. *ViewEL* (e.g., our proposed method): filtering views A and B in Figure 2 are set as “low-pass filtering” and “band-pass filtering with passband of 0.5–40 Hz” [55], respectively. Note that there is no problem if we exchange “low-pass filtering” for “band-pass filtering,” since an effective measure for homogeneous ensemble learning is to make input data different. Here, we just make the 1st path consistent with the research work [6].
2. *AdaBoost*: the filtering view is set as “low-pass filtering.” In the training phase, we utilize the explicit method to train two MCNN[A] models based on the framework of* AdaBoost*. In the testing phase, we apply “subview prediction” to individual classifiers independently and fuse them afterwards.
3. *Bagging*: most of the configuration parameters are the same as those of* AdaBoost*. The only difference is that two MCNN[A] models are obtained based on the framework of* Bagging*.
4. *YeC*: the filtering view is set as “low-pass filtering.” We utilize the explicit method to train one MCNN[A] model for each lead, so there are a total of 8 MCNN[A] models since the incoming ECG recording contains 8 leads. In the testing phase, we first apply “subview prediction” to each individual classifier and then employ the* Bayesian *approach (product rule) to fuse them [54].
5. *YeCRaw*: most of the configuration parameters are the same as those of* YeC*. The only difference is that we replace MCNN[A] with MCNN[B]. Note that neither the local view transformation nor “subview prediction” can be used in this method.

### 3.4. Performance Metrics

We utilize the following metrics [56] to evaluate algorithms: specificity (Sp), sensitivity (Se), geometric mean (GMean), accuracy (Acc), and negative predictive value (NPV), given by

(2) Sp=TNTN+FP,Se=TPTP+FN,GMean=Sp×Se,Acc=TP+TNTP+TN+FP+FN,NPV=TNTN+FN,

where TN and TP are the number of normal and abnormal samples correctly classified, respectively, FN is the number of abnormal samples that are classified as normal, and FP is the number of normal samples that are classified as abnormal. In addition, we also utilize related metrics of the ROC (receiver operating characteristic) curve, including AUC (area under the ROC curve), TPR (the vertical axis of the ROC curve, e.g., “Sp” in this study), and FPR (the horizontal axis of the ROC curve, e.g., “1-Se” in this study).

## 4. Results

In this section, we report experimental results on the CCDD database. There are nine groups of testing samples with different sources, namely, DS1~DS9. Besides presenting the testing results of each group, we will summarize the averages and the standard deviations of performance metrics for each algorithm. The metrics GMean takes into consideration the classification results on both positive and negative classes [57], while the key technology of the classification of normal and abnormal ECG recordings with short duration is to make TPR under the condition of NPV being equal to 95% (*TPR95*) as high as possible [58]. Therefore, we perform the* Wilcoxon* signed ranks test [59] to investigate whether the difference in GMean and* TPR95* achieved by different algorithms is statistically significant. Generally speaking, a *p value* that is less than 0.05 indicates the difference is significant, and the smaller the *p value* is, the more significant the difference is.

### 4.1. Effectiveness Evaluation

We first investigate the contribution of “subview prediction.” From the testing results presented in Tables 1 and 2, we can see that most of the performance metrics are increased and the classification performance is improved regardless of “explicit training” and “implicit training.” From the results of statistical analysis summarized in Tables 3 and 4, we know that “subview prediction” significantly outperforms “simple prediction” in terms of GMean, no matter which training method we use. In addition, we also find that both “explicit training” and “implicit training” have advantages and disadvantages: the former has higher Se while the latter has higher Sp. From the perspective of ensemble learning, these two classifiers are exactly what we want. Next, we present their fusion results in Tables 5 and 6.

It is observed from the testing results presented in Table 5 that the fusion model always has the highest AUC. From the statistical results summarized in Table 6, we can see that the fusion model significantly outperforms the explicit [6] and implicit (the classification models are obtained by “explicit training” and “implicit training,” resp., and the results are based on subview prediction) models in terms of GMean and all other models in terms of* TPR95*. All these findings illustrate the effectiveness of our newly developed generation and fusion strategies.

### 4.2. Comparison with Other Methods

We compare the performance of the proposed method (e.g.,* ViewEL*) with that of* YeCRaw*,* YeC*,* Bagging,* and* AdaBoost*. From the testing results presented in Table 7, we can see that* ViewEL* produced the best results in many of the performance metrics. From the results of statistical analysis summarized in Table 8, we know that* ViewEL* significantly outperforms* YeCRaw* and* Bagging* in terms of both GMean and* TPR95* and* YeC* in terms of GMean. Although we cannot say that* ViewEL* significantly outperforms* AdaBoost* in terms of GMean, the *p*value is a little greater than 0.05.

The essential difference between* YeC* and* YeCRaw* is whether the local view transformation and “subview prediction” are used. Table 7 shows that* YeC* increases almost all the metrics except Sp and significantly outperforms* YeCRaw* in terms of both GMean and* TPR95* (the *p* values are 0.0039 and 0.0156, resp.). Likewise, the performance of* Bagging* and* AdaBoost* will be degraded if we remove the view transformations and “subview prediction” from them, respectively. Of course, their performance could be improved if the number of DNN models is increased. For* ViewEL*, we can also train multiple individual classifiers to enhance performance using different validation samples, such as time subseries starting from other positions and time subseries extracted from some part of training samples in “implicit training.” However, besides classification performance, we should also take into consideration the computational efficiency since the DNN is a model with high complexity. In practical applications (telemedicine centers), an effective ensemble method with less number of individual classifiers is needed.

## 5. Conclusion

The current work proposes a novel DNN-based ensemble method that uses multiple view-related strategies, such as the view transformations, “implicit training,” and “subview prediction.” Experiment results on the CCDD database demonstrate that the proposed method is effective for biomedical time series classification. Furthermore, we compare it with some well-known methods for the classification of normal and abnormal ECG recordings. Our proposed method achieves comparable or better classification results than those by the others.

It is worth noting that this study just presents a new research idea for ensemble learning, and we can incorporate more strategies into Figure 2, such as wavelet-transformation views, compressive sensing view, class-switching technology, and misclassification cost. Those are all research tasks we will do in the future.

## Figures and Tables

**Algorithm 1:**
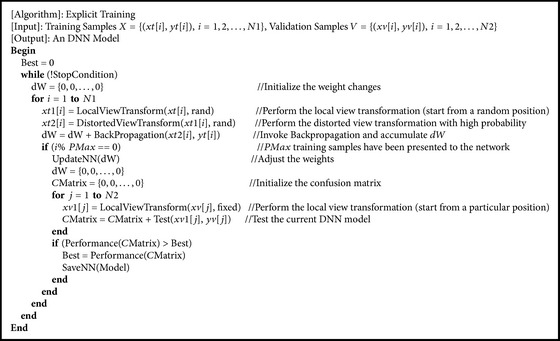
Pseudocode of explicit training.

**Algorithm 2:**
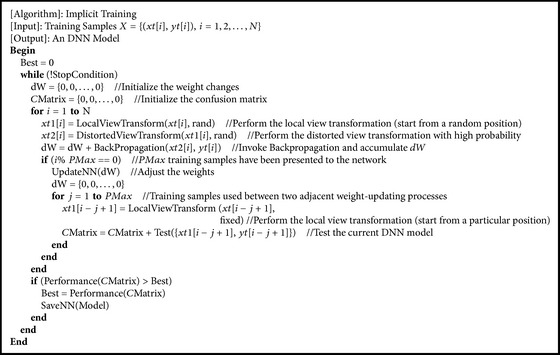
Pseudocode of implicit training.

**Table 1:** Contribution of subview prediction (explicit training).

Dataset	Method	Sp (%)	Se (%)	GMean (%)	Acc (%)	AUC	NPV = 95%	
							TPR (%)	FPR (%)
DS1	Simple [6]	88.84	76.95	82.68	85.41	0.9034	63.322	8.217
	*Subview*	*89.85*	*76.81*	*83.07*	*86.09*	*0.9123*	*71.111*	*9.227*

DS2	Simple [6]	88.63	79.55	83.97	85.99	0.9174	74.389	9.558
	*Subview*	*89.92*	*80.05*	*84.84*	*87.05*	*0.9272*	*78.879*	*10.135*

DS3	Simple [6]	86.58	77.69	82.01	84.03	0.8972	65.597	8.599
	*Subview*	*87.81*	*78.03*	*82.77*	*85.01*	*0.9074*	*72.506*	*9.505*

DS4	Simple [6]	82.75	84.81	83.77	83.91	0.9096	0.091	0.011
	*Subview*	*83.67*	*85.09*	*84.38*	*84.47*	*0.9153*	*0.051*	*0.004*

DS5	Simple [6]	79.52	86.20	82.79	83.23	0.9084	0^*∗*^	0^*∗*^
	*Subview*	*80.70*	*86.64*	*83.62*	*84.00*	*0.9144*	*0*	*0*

DS6	Simple [6]	81.98	84.90	83.43	83.57	0.9101	0.025	0.003
	*Subview*	*82.80*	*85.49*	*84.13*	*84.26*	*0.9169*	*0*	*0*

DS7	Simple [6]	77.81	84.71	81.19	81.28	0.8905	0.010	0.001
	*Subview*	*78.60*	*85.17*	*81.82*	*81.90*	*0.8964*	*0*	*0*

DS8	Simple [6]	78.31	84.74	81.47	81.71	0.8913	0	0
	*Subview*	*79.46*	*85.23*	*82.30*	*82.51*	*0.8976*	*0.020*	*0.003*

DS9	Simple [6]	83.97	75.40	79.57	81.48	0.8661	1.196	0.159
	*Subview*	*84.62*	*76.04*	*80.22*	*82.13*	*0.8778*	*52.160*	*6.722*

^*∗*^We can change the discrimination threshold from 0 to 1 and calculate the corresponding values of Se, Sp, and NPV. As for “0,” it means that the condition of NPV being equal to 95% cannot be satisfied.

**Table 2:** Contribution of subview prediction (implicit training).

Dataset	Method	Sp (%)	Se (%)	GMean (%)	Acc (%)	AUC	NPV = 95%	
							TPR (%)	FPR (%)
DS1	Simple	91.74	73.25	81.97	86.40	0.9047	64.482	8.368
	*Subview*	*92.58*	*73.52*	*82.50*	*87.08*	*0.9149*	*71.202*	*9.239*

DS2	Simple	91.73	76.51	83.77	87.30	0.9228	75.336	9.680
	*Subview*	*92.45*	*76.40*	*84.04*	*87.78*	*0.9318*	*80.983*	*10.405*

DS3	Simple	89.30	74.65	81.64	85.10	0.9030	70.424	9.232
	*Subview*	*90.23*	*74.88*	*82.20*	*85.84*	*0.9112*	*74.256*	*9.735*

DS4	Simple	87.31	82.30	84.77	84.49	0.9131	0	0
	*Subview*	*88.15*	*82.30*	*85.18*	*84.85*	*0.9192*	*0*	*0*

DS5	Simple	85.80	82.18	83.97	83.79	0.9067	0	0
	*Subview*	*86.78*	*82.34*	*84.53*	*84.31*	*0.9135*	*0*	*0*

DS6	Simple	86.98	81.30	84.09	83.90	0.9098	0	0
	*Subview*	*88.21*	*81.60*	*84.84*	*84.62*	*0.9168*	*0*	*0*

DS7	Simple	83.71	80.09	81.88	81.89	0.8872	0.045	0.012
	*Subview*	*84.19*	*80.46*	*82.30*	*82.31*	*0.8943*	*0*	*0*

DS8	Simple	83.53	80.40	81.95	81.87	0.8891	0.101	0.013
	*Subview*	*84.79*	*80.97*	*82.86*	*82.77*	*0.8962*	*0.032*	*0.004*

DS9	Simple	87.07	71.34	78.81	82.51	0.8635	0	0
	*Subview*	*87.24*	*70.91*	*78.65*	*82.51*	*0.8733*	*0*	*0*

**Table 3:** Statistical results of different testing methods (explicit training).

Method	Sp (%)	Se (%)	GMean (%)	*p* value	Acc (%)	AUC	NPV = 95%		*p* value
							TPR (%)	FPR (%)	
Simple [6]	83.16 ± 4.20	81.66 ± 4.20	82.32 ± 1.42	0.0039	83.40 ± 1.68	0.8993 ± 0.02	22.74 ± 33.90	2.95 ± 4.40	0.1953
*Subview*	*84.16 ± 4.28*	*82.06 ± 4.27*	*83.02 ± 1.44*	*—*	*84.16 ± 1.76*	*0.9073 ± 0.01*	*30.53 ± 36.86*	*3.96 ± 4.78*	*—*

**Table 4:** Statistical results of different testing methods (implicit training).

Method	Sp (%)	Se (%)	GMean (%)	*p* value	Acc (%)	AUC	NPV = 5%		*p* value
							TPR (%)	FPR (%)	
Simple	87.46 ± 3.01	78.00 ± 4.15	82.54 ± 1.83	0.0078	84.14 ± 1.91	0.9000 ± 0.02	23.38 ± 35.13	3.03 ± 4.56	0.3125
*Subview*	*88.29 ± 3.00*	*78.15 ± 4.30*	*83.01 ± 2.00*	*—*	*84.68 ± 1.96*	*0.9079 ± 0.02*	*25.16 ± 37.82*	*3.26 ± 4.90*	*—*

**Table 5:** Performance comparison of different classification models.

Dataset	Model	Sp (%)	Se (%)	GMean (%)	Acc (%)	AUC	NPV = 95%	
							TPR (%)	FPR (%)
DS1	Explicit [6]	88.84	76.95	82.68	85.41	0.9034	63.322	8.217
	Explicit^[a]^	89.85	76.81	83.07	86.09	0.9123	71.111	9.227
	Implicit^[a]^	92.58	73.52	82.50	87.08	0.9149	71.202	9.239
	*Fusion*	*91.81*	*74.96*	*82.96*	*86.95*	*0.9172*	*74.063*	*9.611*

DS2	Explicit [6]	88.63	79.55	83.97	85.99	0.9174	74.389	9.558
	Explicit^[a]^	89.92	80.05	84.84	87.05	0.9272	78.879	10.135
	Implicit^[a]^	92.45	76.40	84.04	87.78	0.9318	80.983	10.405
	*Fusion*	*91.47*	*78.27*	*84.61*	*87.64*	*0.9324*	*81.468*	*10.468*

DS3	Explicit [6]	86.58	77.69	82.01	84.03	0.8972	65.597	8.599
	Explicit^[a]^	87.81	78.03	82.77	85.01	0.9074	72.506	9.505
	Implicit^[a]^	90.23	74.88	82.20	85.84	0.9112	74.256	9.735
	*Fusion*	*89.57*	*76.29*	*82.66*	*85.76*	*0.9122*	*74.819*	*9.809*

DS4	Explicit [6]	82.75	84.81	83.77	83.91	0.9096	0.091	0.011
	Explicit^[a]^	83.67	85.09	84.38	84.47	0.9153	0.051	0.004
	Implicit^[a]^	88.15	82.30	85.18	84.85	0.9192	0	0
	*Fusion*	*86.64*	*84.18*	*85.40*	*85.25*	*0.9215*	*0.020*	*0.005*

DS5	Explicit [6]	79.52	86.20	82.79	83.23	0.9084	0	0
	Explicit^[a]^	80.70	86.64	83.62	84.00	0.9144	0	0
	Implicit^[a]^	86.78	82.34	84.53	84.31	0.9135	0	0
	*Fusion*	*84.69*	*84.82*	*84.76*	*84.76*	*0.9187*	*0*	*0*

DS6	Explicit [6]	81.98	84.90	83.43	83.57	0.9101	0.025	0.003
	Explicit^[a]^	82.80	85.49	84.13	84.26	0.9169	0	0
	Implicit^[a]^	88.21	81.60	84.84	84.62	0.9168	0	0
	*Fusion*	*86.31*	*83.81*	*85.05*	*84.96*	*0.9213*	*19.835*	*0.882*

DS7	Explicit [6]	77.81	84.71	81.19	81.28	0.8905	0.010	0.001
	Explicit^[a]^	78.60	85.17	81.82	81.90	0.8964	0	0
	Implicit^[a]^	84.19	80.46	82.30	82.31	0.8943	0	0
	*Fusion*	*82.08*	*83.02*	*82.55*	*82.55*	*0.9000*	*10.769*	*0.561*

DS8	Explicit [6]	78.31	84.74	81.47	81.71	0.8913	0	0
	Explicit^[a]^	79.46	85.23	82.30	82.51	0.8976	0.020	0.003
	Implicit^[a]^	84.79	80.97	82.86	82.77	0.8962	0.032	0.004
	*Fusion*	*82.69*	*83.32*	*83.00*	*83.02*	*0.9011*	*10.889*	*0.514*

DS9	Explicit [6]	83.97	75.40	79.57	81.48	0.8661	1.196	0.159
	Explicit^[a]^	84.62	76.04	80.22	82.13	0.8778	52.160	6.722
	Implicit^[a]^	87.24	70.91	78.65	82.51	0.8733	0	0
	*Fusion*	*86.46*	*73.37*	*79.64*	*82.66*	*0.8806*	*53.151*	*6.850*

^[a]^The classification models are obtained by “explicit training” and “implicit training,” respectively, and the results are based on subview prediction.

**Table 6:** Statistical results of different classification models.

Model	Sp (%)	Se (%)	GMean (%)	*p* value	Acc (%)	AUC	NPV = 95%		*p* value
							TPR (%)	FPR (%)	
Explicit [6]	83.16 ± 4.20	81.66 ± 4.20	82.32 ± 1.42	0.0039	83.40 ± 1.68	0.8993 ± 0.02	22.74 ± 33.90	2.95 ± 4.40	0.0156
Explicit^[a]^	84.16 ± 4.28	82.06 ± 4.27	83.02 ± 1.44	0.1641	84.16 ± 1.76	0.9073 ± 0.01	30.53 ± 36.86	3.96 ± 4.78	0.0156
Implicit^[a]^	88.29 ± 3.00	78.15 ± 4.30	83.01 ± 2.00	0.0039	84.68 ± 1.96	0.9079 ± 0.02	25.16 ± 37.82	3.26 ± 4.90	0.0078
*Fusion*	*86.86 ± 3.51*	*80.23 ± 4.49*	*83.40 ± 1.80*	*—*	*84.84 ± 1.82*	*0.9117 ± 0.02*	*36.11 ± 34.34*	*4.30 ± 4.74*	*—*

^[a]^The classification models are obtained by “explicit training” and “implicit training,” respectively, and the results are based on subview prediction.

**Table 7:** Performance comparison of different ensemble methods.

Dataset	Method	Sp (%)	Se (%)	GMean (%)	Acc (%)	AUC	NPV = 95%	
							TPR (%)	FPR (%)
DS1	YeCRaw	95.61	56.11	73.25	84.21	0.8882	52.663	6.834
	YeC	91.44	69.05	79.46	84.98	0.8965	63.302	8.215
	Bagging	91.68	73.90	82.31	86.55	0.9081	67.005	8.694
	AdaBoost	90.75	75.93	83.01	86.47	0.9116	72.031	9.347
	*ViewEL*	*91.81*	*74.96*	*82.96*	*86.95*	*0.9172*	*74.063*	*9.611*

DS2	YeCRaw	95.35	58.84	74.91	84.74	0.9010	65.363	8.399
	YeC	90.84	70.48	80.01	84.92	0.9028	67.815	8.715
	Bagging	91.11	77.31	83.93	87.10	0.9252	77.739	9.988
	AdaBoost	90.62	79.01	84.62	87.25	0.9255	79.503	10.215
	*ViewEL*	*91.47*	*78.27*	*84.61*	*87.64*	*0.9324*	*81.468*	*10.468*

DS3	YeCRaw	94.22	56.82	73.17	83.51	0.8829	55.944	7.349
	YeC	89.65	69.59	78.99	83.91	0.8962	67.103	8.797
	Bagging	88.91	75.55	81.96	85.09	0.9033	69.965	9.172
	AdaBoost	88.13	76.84	82.29	84.90	0.9025	71.536	9.378
	*ViewEL*	*89.57*	*76.29*	*82.66*	*85.76*	*0.9122*	*74.819*	*9.809*

DS4	YeCRaw	92.51	71.35	81.24	80.57	0.9054	0	0
	YeC	86.87	80.21	83.47	83.11	0.9072	0	0
	Bagging	85.83	83.60	84.70	84.57	0.9140	0	0
	AdaBoost	84.32	84.98	84.65	84.69	0.9172	0.362	0.015
	*ViewEL*	*86.64*	*84.18*	*85.40*	*85.25*	*0.9215*	*0.020*	*0.005*

DS5	YeCRaw	91.03	73.22	81.64	81.13	0.9039	0	0
	YeC	84.29	82.58	83.43	83.34	0.9071	0.184	0.008
	Bagging	83.16	84.57	83.86	83.94	0.9106	0	0
	AdaBoost	81.58	86.10	83.81	84.09	0.9143	0.149	0.007
	*ViewEL*	*84.69*	*84.82*	*84.76*	*84.76*	*0.9187*	*0*	*0*

DS6	YeCRaw	92.53	70.79	80.93	80.72	0.9052	0	0
	YeC	86.75	80.09	83.35	83.13	0.9069	0	0
	Bagging	85.26	83.32	84.29	84.21	0.9134	0.074	0.006
	AdaBoost	83.51	85.10	84.30	84.37	0.9158	0.108	0.007
	*ViewEL*	*86.31*	*83.81*	*85.05*	*84.96*	*0.9213*	*19.835*	*0.882*

DS7	YeCRaw	89.00	71.57	79.81	80.23	0.8885	0	0
	YeC	81.93	81.52	81.72	81.72	0.8944	16.324	0.850
	Bagging	80.77	82.62	81.69	81.70	0.8916	0	0
	AdaBoost	79.46	84.81	82.09	82.15	0.8956	0	0
	*ViewEL*	*82.08*	*83.02*	*82.55*	*82.55*	*0.9000*	*10.769*	*0.561*

DS8	YeCRaw	89.14	70.49	79.27	79.29	0.8879	0	0
	YeC	83.27	80.21	81.72	81.65	0.8910	1.989	0.098
	Bagging	81.28	83.02	82.15	82.20	0.8916	8.551	0.410
	AdaBoost	80.21	84.72	82.44	82.59	0.8954	0.014	0.001
	*ViewEL*	*82.69*	*83.32*	*83.00*	*83.02*	*0.9011*	*10.889*	*0.514*

DS9	YeCRaw	92.57	64.06	77.01	84.31	0.8789	49.524	6.382
	YeC	86.02	76.90	81.33	83.37	0.8849	54.494	7.039
	Bagging	87.42	72.30	79.50	83.03	0.8804	56.669	7.302
	AdaBoost	85.71	74.22	79.76	82.38	0.8825	59.061	7.611
	*ViewEL*	*86.46*	*73.37*	*79.64*	*82.66*	*0.8806*	*53.151*	*6.850*

**Table 8:** Statistical results of different ensemble methods.

Method	Sp (%)	Se (%)	GMean (%)	*p* value	Acc (%)	AUC	NPV = 95%		*p* value
							TPR (%)	FPR (%)	
YeCRaw	92.44 ± 2.41	65.92 ± 7.00	77.91 ± 3.42	0.0039	82.08 ± 2.09	0.8936 ± 0.01	24.83 ± 29.75	3.22 ± 3.85	0.0078
YeC	86.78 ± 3.34	76.73 ± 5.50	81.50 ± 1.73	0.0273	83.35 ± 1.18	0.8985 ± 0.01	30.13 ± 31.97	3.75 ± 4.25	0.1289
Bagging	86.16 ± 3.98	79.58 ± 4.78	82.71 ± 1.65	0.0039	84.26 ± 1.82	0.9042 ± 0.01	31.11 ± 35.36	3.95 ± 4.64	0.0391
AdaBoost	84.92 ± 4.23	81.30 ± 4.73	83.00 ± 1.57	0.0547	84.32 ± 1.77	0.9067 ± 0.01	31.42 ± 37.47	4.06 ± 4.86	0.1289
*ViewEL*	*86.86 ± 3.51*	*80.23 ± 4.49*	*83.40 ± 1.80*	*—*	*84.84 ± 1.82*	*0.9117 ± 0.02*	*36.11 ± 34.34*	*4.30 ± 4.74*	*—*

**Table 9:** Data distribution.

	Dataset	Normal	Abnormal	Total	Source
The training samples	data944–25693	8800	3520	12320	Shanghai, District #1
The validation samples	data944–25693	280	280	560	Shanghai, District #1
The testing samples (DS1)	data944–25693	8387	3402	11789	Shanghai, District #1
The testing samples (DS4)	data25694–37082	4911	6352	11263	Shanghai, District #2
The testing samples (DS2)	data37083–72607	25020	10249	35269	Shanghai, District #3
The testing samples (DS3)	data72608–95829	16210	6508	22718	Shanghai, District #4
The testing samples (DS5)	data95830–119551	10351	12948	23299	Shanghai, District #5
The testing samples (DS6)	data119552–141104	9703	11529	21232	Shanghai, District #6
The testing samples (DS7)	data141105–160913	9713	9831	19544	Shanghai, District #7
The testing samples (DS8)	data160914–175871	6944	7781	14725	Shanghai, District #8
The testing samples (DS9)	data175872–179130	2289	935	3224	Suzhou, District #1

## Appendix

Each ECG recording in the Chinese Cardiovascular Disease Database is approximately 10~20 seconds in duration and contains 12 leads, namely, I, II, III, aVR, aVL, aVF, V1, V2, V3, V4, V5, and V6, where II, III, V1, V2, V3, V4, V5, and V6 are orthogonal, while the remaining four can be linearly derived from them. We obtained these data from different hospitals (i.e., real clinical environment) in Shanghai and Suzhou successively. Cardiologists gave the diagnostic conclusion of each recording, which may contain more than one disease type. We used hexadecimal coding (form of “0xdddddd”) to encode disease types which are divided into three grades, including 12 one-level types (e.g., invalid, normal, sinus rhythm, atrial arrhythmia, junctional rhythm, ventricular arrhythmia, conduction block, atrial hypertrophy, ventricular hypertrophy, myocardial infarction, ST-T change, and other abnormities), 72 two-level types, and 297 three-level types. More details can be seen on our website (http://58.210.56.164:88/ccdd or http://58.210.56.164:66/ccdd).

In numerical experiments, we first throw away the invalid ECG recordings and the ones whose duration is less than 9.625 seconds. Then, a data segment of 9.5 s seconds that only contains eight orthogonal leads is extracted from an ECG recording after ignoring the first 0.125 s. Finally, each recording has 8 × 1900 sampling points at the sampling frequency of 200 Hz. We regard a recording as normal if the diagnostic conclusion is “0 × 020101” or “0 × 020102” or “0 × 01”; otherwise, it is regarded as abnormal.  Table 9 summarizes the detail information.

The recordings from “data 944–25693” used as training samples are as follows: 4520–4584, 4586–4613, 4615– 4652, 4654–4761, 4763–4967, 4969–4972, 4975–5146, 5148, 5151–5279, 5281–5300, 5302–5348, 5350–5540, 5542–5568, 5570–5713, 5715–5777, 5779, 5781–5792, 5794–5974, 5976–6074, 6076–6118, 6120–6127, 6129–6134, 6136–6206, 6208–6281, 6283–6441, 6443–6502, 6504–6538, 6540–6654, 6656–6997, 6999–7005, 7007–7012, 7014– 7060, 7062–7451, 7453–7506, 7508–7531, 7533–7594, 7596–7642, 7644–7732, 7734–7739, 7741–7829, 7831–7887, 7889–7939, 7941–7946, 7948–7956, 7980–8064, 8066–8108, 16556–17045, 17047–17128, 17407–17422, 17424–17454, 17456–17928, 17930–17933, 17935–17955, 17957–18093, 18095–18258, 18260–18441, 18443–18538, 18540–18562, 18565–18642, 18644–18814, 18816–18817, 18819–18984, 18986–19327, 19329–19439, 19441– 19647, 19649–19657, 19659–19852, 19854–20173, 20175–20700, 20702–20881, 20883–21252, 21254–21301, 21303–21586, 21588–21815, 21817–21865, 21867–21889, 21891–21986, 21988–22149, 22151–22226, 22228– 22462, 22464–22623, 22625–22793, 22795–22935, 22937–23032, 23034–23038, 23040–23043, 23045–23067, 23069–23268, 23270–23587, 23589–23611, 23613–23973, 23975–24108, 24110–24646, 24648, 24650–24775, 24777–24820, 24822–24962, 24964–25201, 25203–25282, 25284–25301, 25303–25330, 25332–25457, 25459– 25495, 25497–25606, and 25608–25691.

The recordings from “data 944–25693” used as validation samples are as follows: 4176–4278, 4362–4413, 4415–4519, 7957–7979, 17129–17389, and 17391–17406.

There are nine groups of testing samples, namely, DS1~DS9, which were obtained from hospitals located at different districts in Shanghai and Suzhou. DS1 consists of the remaining recordings from “data 944–25693,” while other groups contain all the valid recordings from the corresponding dataset, respectively.
